# Association of circulating serum free bioavailable and total vitamin D with cathelicidin levels among active TB patients and household contacts

**DOI:** 10.1038/s41598-023-32543-2

**Published:** 2023-04-01

**Authors:** Ester Lilian Acen, William Worodria, David Patrick Kateete, Ronald Olum, Moses L. Joloba, Ashraf Akintola, Mudarshiru Bbuye, Irene Biraro Andia

**Affiliations:** 1 Department of Physiology, School of Biomedical Sciences, College of Health Sciences, Makerere University, Kampala, Uganda; 2 Pulmonary Division, Department of Internal Medicine, Mulago National Referral Hospital, Kampala, Uganda; 3 Department of Immunology and Molecular Biology, School of Biomedical Sciences, College of Health Sciences, Makerere University, Kampala, Uganda; 4 Department of Internal Medicine, School of Medicine, College of Health Sciences Unit, Makerere University, Kampala, Uganda; 5 Medical Research Council, Uganda Virus Research Institute and London School of Hygiene and Tropical Medicine Uganda Research Unit, Entebbe, Uganda; 6 Department of Biomedical Convergence Science and Technology, School of Industrial Technology Advances, Kyungpook National University, Daegu, 41566 Republic of Korea; 7 Makerere Lung Institute College of Health Sciences, Makerere University, Kampala, Uganda

**Keywords:** Immunology, Microbiology

## Abstract

The free hormone hypothesis postulates that the estimation of free circulating 25 (OH)D may be a better marker of vitamin D status and is of clinical importance compared to total vitamin D fraction. The unbound fraction is involved in biological activities since it is able to penetrate into the cell. Studies have shown that cathelicidin/LL-37 inhibits the growth of *Mycobacterium tuberculosis* in a vitamin D-dependent manner and therefore adequate vitamin D is required for its expression. The study aimed to determine the association between serum bioavailable and total vitamin D with LL-37 levels in ATB patients, LTBI, and individuals with no TB infection. This was a cross-sectional study in which bioavailable vitamin D and LL-37 levels were measured using competitive ELISA kits and total vitamin D was measured using electrochemilumiscence and consequently determined their association. The mean (SD) bioavailable vitamin D levels of the study participants were 3.8 ng/mL (2.6) and the median (IQR) of LL-37 levels were 320 ng/mL (160, 550 ng/mL). The mean (SD) of total vitamin D levels was 19.0 ng/mL (8.3) ng/mL. Similar weak correlations were observed between the bioavailable and total vitamin D with LL-37 levels, therefore, deviating from our hypothesis.

## Introduction

Vitamin D deficiency is a prominent risk factor for TB disease worldwide^1–4^.Vitamin D can be obtained in two forms, D2 form is obtained through diet, particularly from mushrooms and yeasts and D3 is found in animal products like meat, milk, egg yoke and fish, and fish oil. Vitamin D3 may also be obtained through skin biosynthesis after sunshine exposure^5^. Its main circulating active metabolite 1, 25 (OH)D is involved in the regulation of antimicrobial activity and therefore important in TB therapy^6^. So far, total vitamin D or 25 (OH)D has been considered a better index for determining vitamin D status due to its longer half-life^5,7–10^. However, the free hormone hypothesis postulates that the estimation of free circulating 25 (OH)D may be a better marker of vitamin D status and is of clinical importance compared to total vitamin D levels because it is the fraction involved in biological activities^11–14^. Bioavailable 25 (OH)D is used to represent free vitamin D and the 10–15% fraction is loosely bound to albumin^9,15^. About 85–90% of total 25 (OH)D is bound to VDBP and 10–15% is loosely bound to albumin and a small fraction remains unbound^10,13^. Free 25 (OH)D is increased and readily available to cells when DBP levels are at low concentrations. Previous studies report that changes in DBP levels and 25 (OH)D binding affinity can lead to higher levels of free 25 (OH)D, even in the absence of total vitamin D levels^16,17^. According to the Endocrine Society, total vitamin D status is classified into three groups: < 20 ng/mL deficient, 21–29 ng/mL deficient, and > 30 ng/mL optimal; or sufficient amounts^18^. The widely studied human AMPs involved in innate and adaptive immunity are defensins and cathelicidin/leucine leucine 37 (LL-37). LL-37 is an effector molecule produced by various cells including circulating immune cells such as neutrophils, monocytes, T cells other cells including epithelial cells and other immune barrier sites. In vitro and in vivo studies have shown that LL-37 inhibits the growth of *MTB* in a vitamin D-dependent manner^19,20^. Accordingly, studies have reported that adequate levels of 25 (OH)D are required for the expression of LL-37^21,22^. According to our systematic review, six studies reported that vitamin D regulates LL-37 expression and that vitamin D deficiency alters this function^21^. Because the free fraction of vitamin D, which enters cells to cause biological effects, has not been studied with the LL-37 molecule, we hypothesize that there is no relationship between bioavailable and total vitamin D with the LL-37 levels among the ATB patients, LTBI, and individuals with no TB infection. This study aimed to determine the association between serum bioavailable and total vitamin D with LL-37 levels in ATB patients, LTBI, and individuals with no TB infection.

## Results

### Social demographic characteristics

A total of 148 participants consisting of 56 newly diagnosed ATB patients, 49 individuals with LTBI, and 43 individuals with no TB infection were included in the study. Of these 95 samples 56 ATB patients, 16 LTBI, and 21 individuals with no TB infection were further selected according to specimen availability for total vitamin D analysis. The median age of the study participants was 28 (IQR 20.0–35.0) years with the majority being females. Both HIV-positive and negative individuals were included in the study. Details of the social demographic characteristics and clinical factors are found elsewhere^23,24^.

### Serum bioavailable and total vitamin D levels among ATB LTBI and those with no TB infection

The overall mean (SD) of bioavailable vitamin D levels of the study participants was 3.8 (2.6) ng/mL According to the reference ranges used in this study, 53 (35.8%) participants had < 1.9 ng/mL, and the majority 46 (31%) were ATB patients. Eighty-one (54.7%) were between 1.92 and 8.8 ng/mL and those with values > 8.8 were 14 (9.5%) participants. The ATB patients had the lowest median bioavailable serum vitamin D levels with a statistical significance of P < 0.01 as shown in Table 1. No statistical significance was noted in the bioavailable vitamin D between the male and female participants. Statistical significance was observed in bioavailable vitamin D levels in HIV-positive and HIV-negative subjects, those with BCG scars and subjects without a scar, and in alcohol users and non-users, Table 1 provides further details. Among age categories, age groups up to 18 years had higher bioavailable levels compared to other categories, although no statistical significance was observed. The mean (SD) of total vitamin D levels was 19.0 (8.3) ng/mL. Statistically low total vitamin D levels were found among the ATB patients compared to other groups shown in Table 2. The details of total vitamin D analysis have previously been reported elsewhere^2^.

**Table 1:** Showing bioavailable, vitamin D median levels among social and clinical factors characteristics.

Participant characteristic	Free and bioavailable vitamin D Median (IQR)	*P-*value
Age (years)		0.44
18 and below	4.05 (2.50, 5.30)	
19–30	2.65 (1.30, 5.30)	
31–40	2.70 (1.20, 6.20)	
Above 40	2.65 (1.75, 4.05)	
Sex		0.97
Female	3.05 (1.40, 5.30)	
Male	2.95 (1.50,5.30)	
TB status		< 0.01
No TB infection	5.30 (3.20, 6.20)	
Latent TB infection	4.20 (2.50, 6.20)	
Active TB	1.30 (1.10, 1.80)	
Alcohol consumption		0.01
No	2.50 (1.35, 4.35)	
Yes	5.00 (1.80, 6.30)	
Smoking		0.73
No	3.20 (1.40, 5.30)	
Yes	2.50 (1.40, 5.10)	
HIV status		< 0.01
Negative	3.40 (1.70, 5.50)	
Positive	1.45 (1.30, 3.20)	
BCG scar		0.02
No	1.90 (1.30, 5.10)	
Yes	3.55 (1.80, 5.70)	

Free vitamin D in ng/mL, P-value is < 0.05.

**Table 2:** Shows the mean bioavailable vitamin D levels among TB patients, LTBI, and those with no TB infection.

TB status	Frequency (n)	Bioavailable (vitamin D ng/mL) Mean (SD)	*P* value	Total vitamin D (ng/mL) Mean (SD)	*P* value
ATB	56	1.9 (1.8)	< 0.01	17.0 (7.6)	< 0.01
No TB infection	43	5.1 (2.3)		22.0 (7.1)	
LTBI	49	4.7 (2.5)		23.0 (9.4)	
TOTAL	148	3.8 (2.6)		19.0 (8.3)	

*SD* standard deviation, *LTBI* latent TB infection.

### Correlation of bioavailable and total vitamin D levels in ATB, LTBI, and those with no TB infection

**Figure 1:**
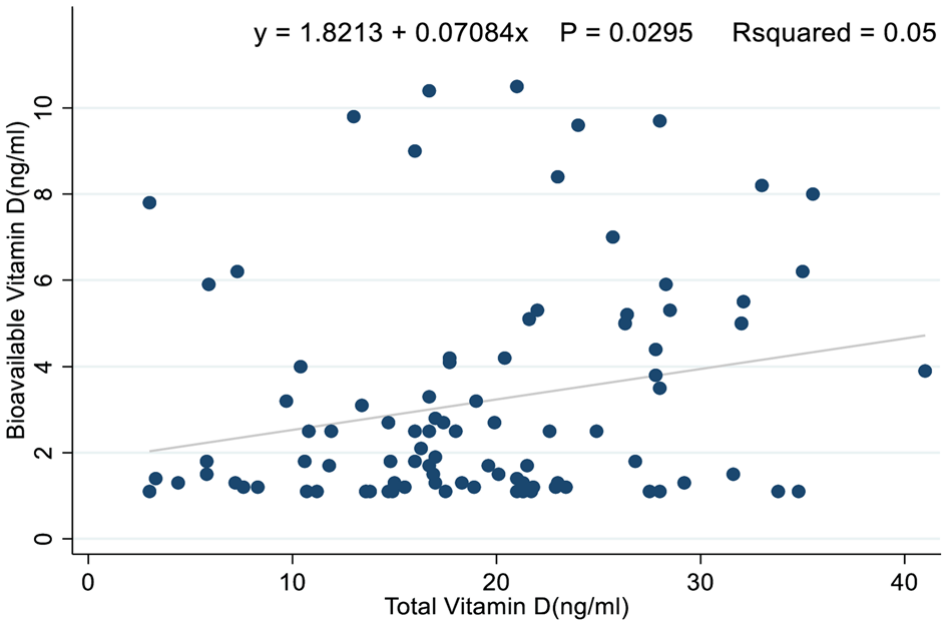
Correlation of bioavailable total vitamin D levels in ATB, LTBI, and individuals with no TB infection.

An analysis of the relationship between bioavailable and total vitamin D levels was performed and a significantly weak positive association was found, r = 0.2, P = 0.03. Other details of the analysis are shown in Fig. 1.

### Serum LL-37 levels among ATB patients, LTBI, and individuals with no TB infection

**Figure 2:**
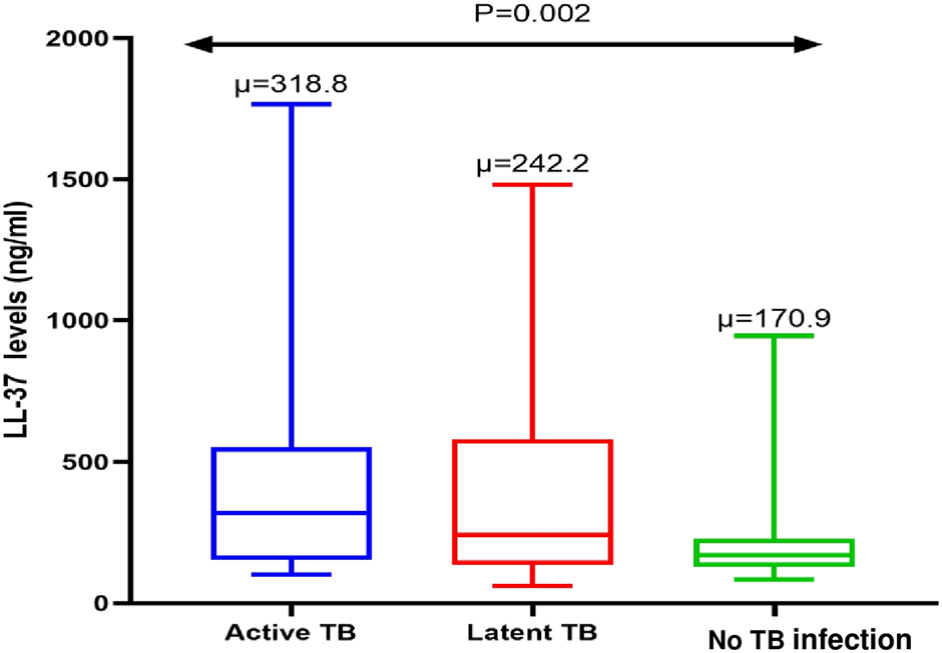
Comparison of serum LL-37 levels among ATB patients, LTBI individuals, and individuals with no TB infection. Median serum concentrations were significantly higher among the ATB patients compared to the LTBI and those with no TB infection.

An analysis of LL-37 levels was performed and the median (IQR) was 320 ng/mL (160, 550 ng/mL). Higher LL-37 levels were found among the ATB compared LTBI and those with no infection TB groups, P ≤ 0.05 as shown in Fig. 2. Other details of the LL-37 analysis have been reported elsewhere^23^.

### Correlation of LL-37 with bioavailable vitamin D levels in ATB patients, LTBI, and those with no TB infection

**Figure 3:**
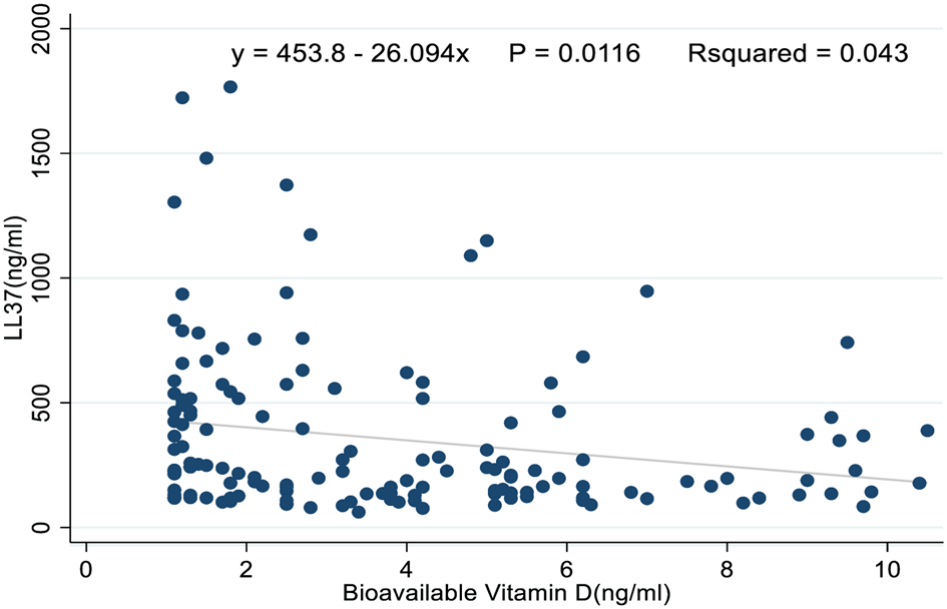
Shows the correlation of LL-37 with Bioavailable vitamin D levels among ATB patients, LTBI, and individuals with no TB infection.

A correlation of LL-37 with bioavailable vitamin D levels between the three groups was performed and a significantly weak negative association was observed r = − 0.2, P = 0.01. Figure 3 shows the details.

### Correlation of LL-37 and total vitamin D levels in ATB patients, LTBI, and those with no TB infection

**Figure 4:**
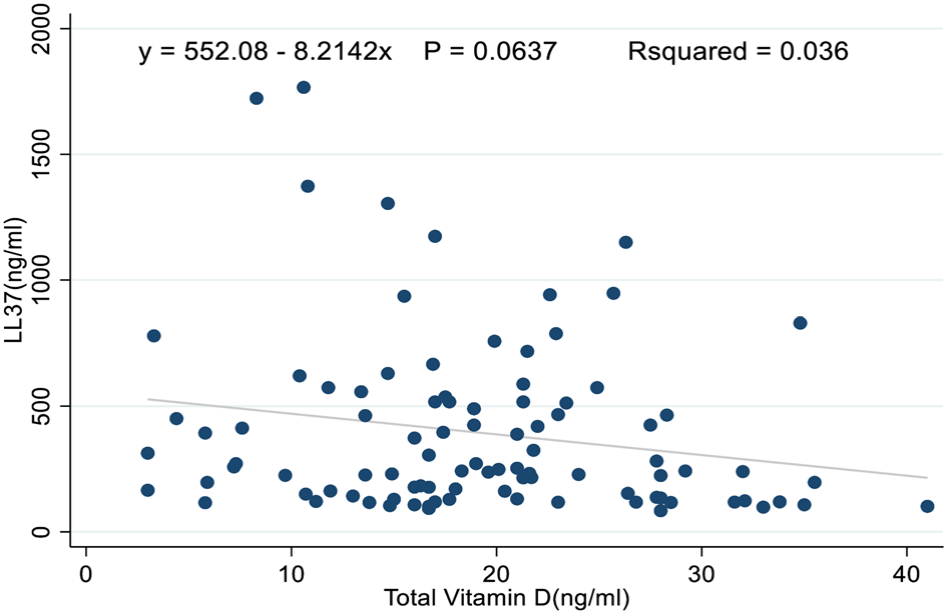
Correlation of LL-37 with total vitamin D levels in ATB, LTBI, and individuals with no TB infection.

A correlation between LL-37 and total vitamin D was performed and overall a statistically significant weak negative association was found, r =  − 0.2, P = 0.04 and details are shown in Fig. 4. When the analysis was performed between the two molecules in the group with adequate vitamin D levels, this was a very weak positive result and an insignificant association was observed, r = 0.01 *P* = 0.98.

## Discussion

High LL-37 levels and low vitamin D levels were found among the ATB patients compared to the LTBI patients and those with no TB infection. These results are comparable to our systematic review, which high levels of LL-37 found and low levels of vitamin D in TB patients compared to the controls^25^. Both the bioavailable levels and total vitamin D levels were lower among TB patients compared to other study groups. We performed a correlation between total and bioavailable vitamin D levels and a significantly weak positive correlation was observed. These findings are comparable to a previous study that performed the same correlation and found a positive association, although that study reported a stronger association compared to the present study^26^. In another recent study the bioavailable and total vitamin D among older adults mentioned that these two fractions highly correlate although the even when the bioavailable levels fraction is very low^23^. The association performed between bioavailable and total vitamin D levels with LL-37 showed weak associations for both fractions. This observation may further reflect that the bioavailable levels correlating with the total fraction and therefore may not be a better indicator of vitamin D status in TB disease. Additionally, our finding is similar to a recent study that performed the same correlation in pregnant women^10^. Furthermore, findings from another study showed the same correlation in postmenopausal women in American and African American populations, and no difference was found by race^24^. According to the study by Naweed et al. the same finding was reported about race for both bioavailable and total vitamin D levels^13^. Generally, all these studies concluded that free vitamin D levels are not superior to total vitamin D and may not be a better index of vitamin D status. On the contrary, two studies reported that free vitamin D levels may be a better predictor of vitamin status than total levels based on the association observed^27,28^. The study by Bhan et al. found a positive association with vitamin D levels above 30 ng/mL^29^. On the contrary, our study found a weak association between the sufficient groups. However, a stronger association was observed in the correlation analysis of the bioavailable vitamin D with LL-37 levels in the group with optimal vitamin D levels. This finding is possibly caused by the action of free 1,25-dihydroxy vitamin D, the bioactive molecule that regulates LL-37. Although the bioavailable levels may not be a better index of vitamin D status compared to total vitamin D this scenario can suggest that the free fraction of vitamin D may be more efficient in the production of free 1,25-dihydroxy vitamin and therefore better in regulating LL-37 expression compared to total levels. A study by Johnsen et al. found a stronger correlation between free vitamin D and total vitamin D levels and bone mineral density^30^. According to Aloia et al., reference ranges for free vitamin D levels may not be relevant due to racial differences, and also these levels may depend on vitamin D status rather than hormonal control^24^. The other studies that found free vitamin D levels to be better than total vitamin D levels included a better association with physical activity in older African-American women^27^, better survival in colorectal cancer patients^28^ better indicator in patients with hepatocellular carcinoma^31^ and the one who found an association between free vitamin D and the risk of mortality in patients with coronary heart disease^32^ could be a consequence of changes in the DBP levels. Conditions that modify the DBP levels and its binding affinity with vitamin D metabolite cause these alterations^33^. In diseases such as liver cirrhosis and liver failure due to malabsorption of proteins, there is a reduction in DBP and albumin. This in turn leads to a reduction in the binding affinity of vitamin D and thus an increase in the level of free and bioavailable vitamin D. On the other hand, poor transport of the DBP in renal failure leads to a reduction in the protein and thus to the same changes^33^. In general, DBP gene variants control the amount of circulating DBP and alter binding affinity, free and bioavailable levels^34,35^. The DBP therefore plays a crucial role in the outcome of vitamin D status through its function as a reservoir of total vitamin D and as a regulator of free and bioavailable levels^36^. Accordingly, the Gc1F genotype, which is predominantly found in the African population, is believed to have a low concentration of the DBP gene and higher levels of free and bioavailable vitamin^37^. These inconsistent findings necessitate further research to unfold substantial insights.

To our knowledge, this was the first study to perform an analysis of the bioavailable vitamin D levels containing the LL-37 molecule in TB patients. The differences between the male and female free vitamin D levels were not statistically significant although the female had higher levels. This may probably be due to the difference in the amount of DBP levels between them.

Regarding age, the younger participants had higher bioavailable vitamin D levels compared to the other age groups although no statistical significance was noted. As reported earlier the total vitamin D also reported no significance with age in the study groups^38^.

According to the free hormone hypothesis, the effective and clinically important fraction of vitamin D is the 10–15% that enters the cells^11^. This part may be responsible for vitamin D immunomodulation in numerous disease states, including TB. However, vitamin D bioavailability can be controlled by several factors involved in its absorption, transport, and metabolism^39^. Furthermore, according to Mendel, the movement of the hormone into the cell depends on the separation of this hormone from its binding protein, blood flow rate, and absorption into the cell^40^. According to our systematic review, previous studies have performed analyses between total vitamin D and LL-37 levels among TB patients^41–43^. To our knowledge, this is the first study to examine the relationship between bioavailable vitamin D and LL-37 levels in TB patients. The few studies found have evaluated bioavailable vitamin D and LL-37 levels in other disease states^38,44,45^.

In general, an accurate interpretation of bioavailable vitamin D levels may require an estimate of DBP levels, which can act as confounders. According to Bhan, low DBP levels were found in a healthy black population, and another study on pregnant women reported the same finding^46^. Consequently, the bioavailable vitamin D levels in the black population are expected to be higher than in other populations that have high DBP levels.

We recognize that one of the limitations of the present study is the lack of estimation of serum DBP levels, which represent the main transport of 25 (OH) D. Another limitation was that the correlation of bioavailable vitamin D levels with bone mineral density was not measured. We were unable to measure PTH in our study. According to previous studies, a correlation between PTH and vitamin D levels is an indicator of good bone mineral density.

The strength of this study is the direct measurement of the bioavailable vitamin D levels using the competitive ELISA method, which gives accurate results compared to the indirectly calculated methods.

## Conclusion

Significantly weak inverse associations were found between the bioavailable and total vitamin D with LL-37 levels. Therefore vitamin D is involved in the regulation of LL-37 expression and low vitamin D levels can alter this relationship. Studies on the correlation of bioavailable vitamin D and 1,25dihydroxivtamin D and LL-37 are warranted to confirm our results.

## Methods

### Study design study site and study participants

A comparative cross-sectional study of newly diagnosed ATB patients, LTBI, and individuals with no TB infection aged between 12 and 65 years was conducted. ATB patients were enrolled between the periods July 2019 to August 2020 and the LTBI, and samples from non-TB-infected individuals from the KTB project were used.

### Laboratory analysis

#### Measurement bioavailable vitamin D using the ELISA method

Free serum 25 (OH)D was measured using a 96-well competitive (ELISA) kit catalogue, abx570015 (abbexa) Ltd., Cambridge, UK. The inter-assay and intra-assay CVs were less than 10%. The sensitivity of the assay was 1.88 ng/mL and the minimum detection range was between 3.125 200 ng/mL. The diluted standards and the control were pipetted into the standard and control wells. The plate was placed on a shaker to mix gently. The detection reagent working solution was added to each well and the plate was placed on the shaker to mix. The plate was covered with a seal and incubated at 37 °C for 45 min. The solution was discarded. Using a 300 L multi-channel pipette, the plate was filled with wash buffer and washed three times. After washing, the remaining wash buffer was removed by decantation. The working solution of Detection Reagent B was added to each well. The plate was sealed and incubated at 37 °C for 30 min. The solution was discarded and the wash step was repeated as before. The 3,3,5,5-tetramethylbenzidine (TMB) substrate was added to each well. The plate was covered with a seal and placed on a shaker to mix and was incubated for 10 min at 37 °C, forming a blue color. A stop solution was added to each well and mixed thoroughly, the solution turned yellow. The OD was immediately measured at 450 nm using a spectrophotometer. The intensity of the yellow color was inversely related to the amount of vitamin D bound on the plate. A standard curve was constructed and a best-fit trend line was fitted through the standard points with an R2 of 0.97. A reference range of 1.9–8.8 ng/mL, adopted from Pathology Associates Medical Laboratories (PAML), was used.

#### Measurement of total vitamin D using electrochemiluminescence

Total vitamin D levels were analyzed by electrochemiluminescence using Elecsys vitamin D3 assay according to the manufacturer’s instructions. The assay was performed in three incubation steps.

#### Measurement of LL-37 using the ELISA method

A human 96-well competitive enzyme-linked immunosorbent assay (ELISA) kit catalog (CAMP), abx150919 (abbexa Ltd, Cambridge, UK) was used to determine LL-37 according to the manufacturer’s instructions.

### Statistical analysis

Data were analyzed using STATA software (Stata Corp. STATA Version 16.0, College Station, Texas, USA and Graph Pad Prism (Version 8). Normal distribution was calculated using the Shapiro–Wilk, Anderson–Darling, D’Agostino, and Pearson tests tested and Kolmogorov–Smirnov tests. Continuous data were analyzed in medians and interquartile range (IQR), and means and standard deviation (SD) confidence interval (CI) at 95% and alpha of P < 0.05 was considered significant, and power of 80%. Categorical variables were summarized as n (%) Mann–Whitney U test was used for variables with two categories and the Kruskal–Wallis test for 3 or more categories. Correlations between LL-37 and vitamin levels D levels were performed using pairwise correlation. A reference range of 1.92–8.82 ng/mL adopted by Pathology Associates Medical Laboratories (PAML) was used in the study (Supplementary Information).

### Ethics approval and consent to participate

The present study was conducted according to the Helsinki Declaration. The study was approved by Makerere University School of Biomedical Sciences Higher Degree Research and Ethics Committee (SBS HDREC) (#SBS-637), Research and Ethics Committee Mulago Hospital, Kiruddu Referral Hospital, and the National Council of Science, and Technology (HS2639). All experimental protocols were approved by Makerere University SBS HDREC (#SBS-637), and the National Council of Science and Technology (HS2639). A waiver of consent was sought to use the KTB samples. Written informed consent was obtained from the active TB patients and personal information was kept confidential by using serial codes with no names recorded on the questionnaire. All adult participants of the KTB study gave written informed consent for participation. Additionally Informed consent was obtained from the parents or guardians of the minors.

## Supplementary Information

Supplementary Information.

## Data Availability

All data and reagents are available on request by the corresponding author.

## Abbreviations

ATB: Active TB
DBP: D binding protein
ELISA: Enzyme-linked immunosorbent assay
HIV: Human immunodeficiency virus
IOR: Interquartile range
KTB: Kampala TB cohort
LL37: Cathelicidin
LTBI: Latent TB infection
MTB: Mycobacterium tuberculosis
PTH: Parathyroid hormone
TB: Tuberculosis
